# Supplementary material for: Heat adaptation of phage T7 under an extended genetic code
Source: Virus Evol. 2021 Dec 1;7(2):veab100. doi: 10.1093/ve/veab100 (PMC8923235; doi:10.1093/ve/veab100)
Supplement: veab100_Supp [file veab100_supp.zip › Sequencing_Analysis.zip!/Sequencing_Analysis/A2/indexA2.html]

BRESEQ :: Mutation Predictions


|  |  |
| --- | --- |
|  | ***breseq***  version 0.30.1    mutation predictions \| marginal predictions \| summary statistics \| genome diff \| command line log |

| Predicted mutations | | | | | |
| --- | --- | --- | --- | --- | --- |
| evidence | position | mutation | annotation | gene | description |
| MC JC | 1,257 | Δ1,480 bp | intergenic (–/–) | – / – | –/– |
| RA | 15,094 | G→T | intergenic (–/–) | – / – | –/– |
| RA | 18,406 | G→A | intergenic (–/–) | – / – | –/– |
| RA | 19,721 | G→A | intergenic (–/–) | – / – | –/– |
| RA | 20,544 | G→A | intergenic (–/–) | – / – | –/– |
| RA | 21,276 | A→G | intergenic (–/–) | – / – | –/– |
| RA | 24,161 | A→C | intergenic (–/–) | – / – | –/– |
| RA | 24,238 | A→G | intergenic (–/–) | – / – | –/– |
| RA | 27,932 | T→C | intergenic (–/–) | – / – | –/– |
| RA | 28,691 | A→C | intergenic (–/–) | – / – | –/– |
| RA | 29,258 | A→G | intergenic (–/–) | – / – | –/– |
| RA | 29,697 | C→A | intergenic (–/–) | – / – | –/– |
| RA | 34,975 | A→G | intergenic (–/–) | – / – | –/– |
| RA | 35,095 | A→G | intergenic (–/–) | – / – | –/– |
| RA | 37,346 | T→C | intergenic (–/–) | – / – | –/– |

| Unassigned missing coverage evidence | | | | | | | | | | |
| --- | --- | --- | --- | --- | --- | --- | --- | --- | --- | --- |
|  |  |  | seq id | start | end | size | ←reads | reads→ | gene | description |
| \* | \* | ÷ | NC\_001604.1 | 1 | 111 | 111 | NA [0] | [225] 233 | *–/–* | –/– |
| \* | \* | ÷ | NC\_001604.1 | 13456 | 14296 | 841 | 282 [2] | [3] 241 | *–/–* | –/– |
| \* | \* | ÷ | NC\_001604.1 | 39743 | 39937 | 195 | 241 [230] | [0] NA | *–/–* | –/– |

| Unassigned new junction evidence | | | | | | | | | | | |
| --- | --- | --- | --- | --- | --- | --- | --- | --- | --- | --- | --- |
|  | | seq id | position | reads (cov) | reads (cov) | score | skew | freq | annotation | gene | product |
| \* | ? | NC\_001604.1 | = 13277 | 205 (0.450) | 231 (0.520) | 119/586 | 1.3 | 69.5% | intergenic (–/–) | *–/–* | –/– |
|  | ? | NC\_001604.1 | 14297 = | 3 (0.010) |  |  |  |  | intergenic (–/–) | *–/–* | –/– |
| \* | ? | NC\_001604.1 | = 13455 | 2 (0.000) | 269 (0.600) | 158/586 | 0.3 | 94.6% | intergenic (–/–) | *–/–* | –/– |
|  | ? | NC\_001604.1 | 14331 = | 29 (0.070) |  |  |  |  | intergenic (–/–) | *–/–* | –/– |
| \* | ? | NC\_001604.1 | = 27753 | 287 (0.670) | 183 (0.420) +CGCTATATCTGG | 105/544 | 1.5 | 38.0% | intergenic (–/–) | *–/–* | –/– |
|  | ? | NC\_001604.1 | 27754 = | 318 (0.700) |  |  |  |  | intergenic (–/–) | *–/–* | –/– |
